# *In Vivo* Phenotyping for the Early Detection of Drought Stress in Tomato

**DOI:** 10.34133/2019/6168209

**Published:** 2019-11-27

**Authors:** Michela Janni, Nicola Coppede, Manuele Bettelli, Nunzio Briglia, Angelo Petrozza, Stephan Summerer, Filippo Vurro, Donatella Danzi, Francesco Cellini, Nelson Marmiroli, Domenico Pignone, Salvatore Iannotta, Andrea Zappettini

**Affiliations:** ^1^Institute of Materials for Electronics and Magnetism (IMEM), National Research Council (CNR), Parco Area delle Scienze 37/A, 43124 Parma, Italy; ^2^Institute of Bioscience and Bioresources (IBBR), National Research Council (CNR), Via Amendola 165/A, 70126 Bari, Italy; ^3^Università degli Studi della Basilicata, Dipartimento delle Culture Europee e del Mediterraneo: Architettura, Ambiente, Patrimoni Culturali (DICEM), Via S. Rocco, I-75100 Matera, Italy; ^4^ALSIA Centro Ricerche Metapontum Agrobios, s.s. Jonica 106 ,km 448, 2, Metaponto, MT 75010, Italy; ^5^Department of Chemistry, Life Sciences and Environmental Sustainability, University of Parma, Parco Area delle Scienze, 11/A, 43124 Parma, Italy

## Abstract

Drought stress imposes a major constraint over a crop yield and can be expected to grow in importance if the climate change predicted comes about. Improved methods are needed to facilitate crop management via the prompt detection of the onset of stress. Here, we report the use of an in vivo OECT (organic electrochemical transistor) sensor, termed as bioristor, in the context of the drought response of the tomato plant. The device was integrated within the plant's stem, thereby allowing for the continuous monitoring of the plant's physiological status throughout its life cycle. Bioristor was able to detect changes of ion concentration in the sap upon drought, in particular, those dissolved and transported through the transpiration stream, thus efficiently detecting the occurrence of drought stress immediately after the priming of the defence responses. The bioristor's acquired data were coupled with those obtained in a high-throughput phenotyping platform revealing the extreme complementarity of these methods to investigate the mechanisms triggered by the plant during the drought stress event.

## 1. Introduction

Drought is one of the most frequently occurring and damaging abiotic constraints compromising plant growth and crop yield [1, 2]. The increasing demand for water, driven by the need to feed a growing world population, will be exacerbated by reductions in soil water reserves driven by the predicted rise in global temperature. Maintaining crop yields in an environment where drought is even more prevalent than at present is recognized as an urgent priority [3]. Despite this, however, the genetic and physiological basis of a crop yield under conditions of water insufficiency remains inadequately understood [3, 4], largely because simulating a stress which varies both in time and in intensity is very difficult. The plant response to drought stress depends heavily on the duration and severity of the stress but is also influenced by the plant's genotype and its developmental stage [2, 3]. Nevertheless, it is clear that the main consequences of the stress are to reduce the rate of cell division and expansion, which results in the plants forming smaller leaves, shorter stems, and a reduced root system. In a drying soil, nutrient uptake is compromised by the altered physiochemical status and flux of the xylem sap [5].

A number of phenotyping platforms have been explored to characterize the plant drought stress response. These include the use of optical sensors designed to monitor the plants' photosynthetic activity [6–8], growth status [9, 10], and overall water content [11]. While the major focus has remained on the aerial part of the plant, the importance of phenotyping the root system has also been recognized [12]. Continuous monitoring in real time, however, remains the exception rather than the rule, and most commonly, the plant's physiological status is measured only indirectly [13, 14]. A recent development has featured a graphene sensor able to monitor in real time the transport of water from a plant's roots to its leaves [15], while an integrated electrochemical chip-on-plant was used to detect gene expression under stress condition in tobacco leaves [16]. A different class of sensor, referred to as a “bioristor,” has been shown to be able to detect, in vivo and in real time, the changes in the composition of the plant sap in a growing tomato (*Solanum lycopersicum*) plant [17], without interfering with plant functions. The bioristor is based on an organic electrochemical transistor (OECT) realized on textile thread [18, 19] and enables measuring the changes in ion concentration in the plant sap. The research activities regarding OECT are very active, and several reviews have already been published on OECTs [20–22] or on OECTs applied to biology [23–25]. The choice of tomato, a crop which is produced and consumed worldwide, reflects its particular sensitivity to drought stress, especially during its flowering and fruit enlargement phases [1, 26]. Here, a demonstration is given of how the bioristor concept can be exploited as a tool to achieve the early detection of drought stress in tomato applicable also as a complemental tool for plant phenotyping.

## 2. Experiment

### 2.1. Tomato Plants and the Imposition of Drought Stress

**Figure 1:**
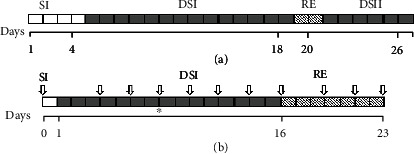
Schematic illustration of the experiments conducted in (a) Parma and (b) Metaponto. White blocks indicate days during which full watering was provided following the bioristor's implantation (SI), grey blocks indicate days during which watering was withheld (DSI, DSII), and the shaded blocks indicate the recovery phase (RE). Arrows indicate the timing of the Scanalyzer readings and image acquisition, while the black star shows when the emergency irrigation was provided.

Two experiments in controlled conditions have been performed. A pilot experiment, carried out in Parma (Italy), was set up to demonstrate the ability of the bioristor to respond to drought stress. The seeds were kindly furnished by ALSIA Metapontum Agrobios Research Center. Seven cv. Red Setter plants were grown up to the stage of 5^th^ fully expanded leaves in 1.5 dm^3^ soil-filled pots under controlled conditions, namely, a constant temperature of 24°C, a relative humidity of 50%, and a 16 h photoperiod. The plants were kept fully irrigated until their fifth true leaf had fully expanded, after which a bioristor was inserted in the stem of each plant (Figure 1(a)). After 3 d, four of the plants were exposed to drought stress by withholding watering for 14 d (DSI); the plants were then irrigated over 2 d (RE), and, finally, a 6 d stress episode was imposed by withholding water (DSII) (Figure 1(a)). A set of four plants was kept fully watered as the control.

On the basis of the results obtained in the pilot experiment, the main experiment was performed in the ALSIA plant phenomics facility (Metaponto, Italy). The trial was based on eight cv. Ikram plants, available at the ALSIA Metapontum Agrobios Research Center, grown in 3 dm^3^ pots exposed to a 12 h photoperiod (light intensity 180 *μ*mol m^−2^ s^−1^) with a daytime temperature of 24°C and a nighttime one of 18°C; the relative humidity ranged from 50 to 60%. When the plant reached the stage of 5^th^ fully expanded leaves, the sensors were integrated and 1 d after the implantation of the bioristor, watering was withheld from four of the plants for 16 d (DSI) and then restored for a further 7 d (RE) (Figure 1(b)). A limited irrigation 50 cm^3^ was supplied to the 3 dm^3^ pots to maintain the plant turgor and allow for the acquisition of images (day 8). The remaining four plants were kept fully watered as the control.

### 2.2. Bioristor Measurements

**Figure 2:**
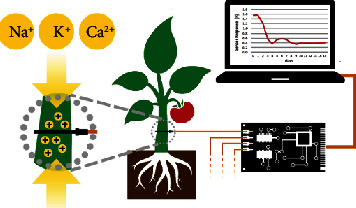
The bioristor setup. The device was inserted into the stem of each tomato plant and connected to a readout system and a computer.

The sensors were prepared, inserted into the plant stems, and connected to a computer, following Coppedè et al. [17] (Figure 2, Supplementary Fig. 1). A constant voltage (*V*_ds_) was applied across the main transistor channel (a textile fiber functionalized with PEDOT:PSS), along with a positive voltage at the gate (*V*_g_); the resulting currents (*I*_ds_) were monitored continuously (for 26 d in the pilot experiment and for 23 d in the main experiment). The sensor response parameter (*R*) was given by the expression (*I*_dS_ − *I*_dS0_)/I_dS0_, where *I*_ds0_ represented the current across the channel when *V*_g_ = 0.

The normalized sensor response (NR) was shown as the bioristor output and calculated as the ratio between *R* of stressed and nonstressed plants. The sensor response parameter *R* was transformed into a normalized sensor response (NR) from the ratio between *R* of stressed and nonstressed plants.

In vitro analyses of the bioristor ability to monitor concentrations in the changes of main cations involved in the drought stress response have been performed. Transfer characteristics of the sensor response were measured using different concentrations of sodium (Na^+^), potassium (K^+^), calcium (Ca^2+^), and magnesium (Mg^2+^) salts expressed as the ratio (*I*_dS_ − *I*_dS0_)/*I*_dS0_ representing the sensor response, where *I*_ds_ is the off current (measured for gate voltages, *V*_g_ ≠ 0V) and *I*_0_ is the on current (measured for *V*_g_ = 0V).

### 2.3. Leaf Stomatal Conductance

Leaf stomatal conductance was measured from two fully expanded leaves per plant (fourth and fifth leaf) of the cv. Ikram plants, using an SC-1 leaf porometer (Decagon Devices, Pullman, WA, USA).

### 2.4. Physiological Analyses

Four controls and four stressed plants have been analyzed for the relative water content (RWC) as reported by Barrs and Weatherley [27], by taking the fully expanded leaf as the sample (two replicates for each plant). Chlorophyll content measurements were performed by using the SPAD 502 meter (Konica Minolta, Ramsey, USA). Measurements from 10 leaves of each plant of varying age and colour were selected for measurements made under diffuse lighting. The relative SPAD value was considered. All data were analyzed statistically applying Student's *t*-test, and the standard error was calculated between replicates.

### 2.5. Nondestructive Phenotyping

Images were captured at 2 d intervals from cv. Ikram plants following Petrozza et al. [9], using a Scanalyzer 3D device (LemnaTec GmbH, Aachen, Germany). The imaging, initiated prior to the imposition of drought stress, involved three mutually orthogonal vantage points, using near-infrared (NIR) and white (RGB) illumination. The NIR images were used to evaluate the plants' water content; the RGB ones were for the assessment of both the plants' state of health (green: healthy tissue, yellow: chlorotic tissue, and brown: necrotic tissue) and for morphological measurements; these data were used to calculate the plants' biovolume and height; their biovolume (a parameter proportional to the aerial mass of the plant) was calculated from the expression [Σ_pixelsideview0°_ + Σ_pixelsideview90°_ + log_10_Σ_pixelsideview_]/3, following Eberius and Lima-Guerra [28] and Petrozza et al. [9]. Plant compactness, which describes how much of the hull area is covered by leaves, was calculated as object area/convex hull area [29]. The NIR index was calculated as weighted mean from the pixel intensities of greyscale NIR images divided into 128 bins, representing a range in leaf water content, while the green index that expresses the fraction of green colour detected in the leaves was calculated in accordance with Casadesús et al. [30].

## 3. Results

### 3.1. The Bioristor Output

**Figure 3:**
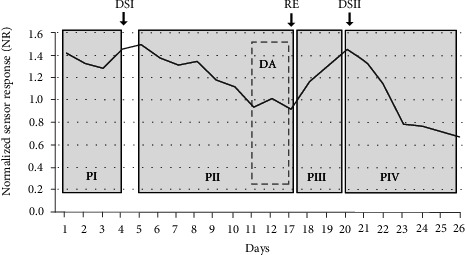
The behaviour of the averaged normalized sensor response parameter (NR) used to monitor the drought response of cv. Red Setter plants subjected to two cycles of drought stress (DSI and DSII) interrupted by a rewatering recovery phase (RE). Arrows show the timing of the initiation of each treatment. The data represent the mean of three well-watered (control) and four stressed plants. The various phases have been highlighted by grey boxing: PI—sensor insertion, PII—drought stress, and PIII—post-rewatering recovery.

**Figure 4:**
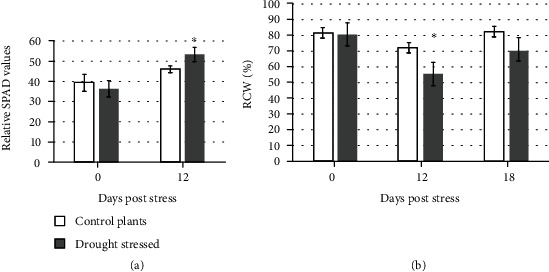
Physiological analyses performed on four drought-stressed plants (dark grey) and the regularly irrigated controls (light grey). (a) SPAD. (b) Relative water content (RWC). Asterisks (∗) indicate significant differences of the drought-stressed plants from the control plants, according to Student's *t*-test (*p* ≤ 0.05).

The monitoring over 26 d of the behaviour of four drought-stressed and three well-watered cv. Red Setter plants confirmed the bioristor's capacity to record day/night changes in the xylem sap's composition [17]. The *R* parameter (analyzed with MATLAB (https://uk.mathworks.com/) and Microsoft Excel 2016) fell during the daytime and rose during the nighttime, as was expected (data not shown). When each 24 h cycle of data was averaged to generate a mean NR value, four distinct phases (defined by a slope change) were recognized (Figure 3): the first phase (PI) covered the first 3 d following the sensor's implantation; PII was initiated 24 h after the withholding of water and was characterized by a decrease in NR over a 6 d period, followed by a short 2 d innate recovery (DA). PIII reflected the plants' recovery following rewatering, during which time NR rose back to its prestress level; finally, during PIV, when the plants were once again deprived of water, NR fell, this time more rapidly than it did during PII. Measurements of both leaf chlorophyll content (as estimated using a SPAD device, Figure 4(a)) and relative water content (RWC, Figure 4(b)) confirmed that the plants were experiencing drought stress (Figure 4(b)). The SPAD value in the stressed plants significantly increases during the drought stress (*p* ≤ 0.05) although only two points have been acquired, and, as expected, the RWC consistently decreases by about 23% (*p* ≤ 0.05) during the drought stress and is completely restored when the recovery occurred (Figure 4(b)).

**Figure 5:**
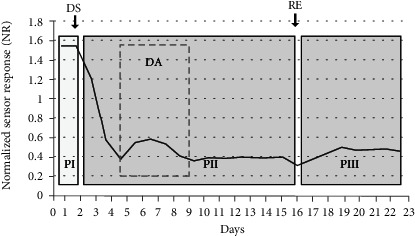
The behaviour of the averaged normalized sensor response parameter (NR) used to monitor the drought response of cv. Ikram plants subjected to drought stress. DS: period during which water was withheld; RE: rewatering recovery phase. The data represent the mean of four well-watered (control) and four stressed plants. The various phases have been highlighted by grey boxing: PI—sensor insertion, PII—drought stress, DA—drought avoidance, and PIII—post-rewatering recovery.

**Figure 6:**
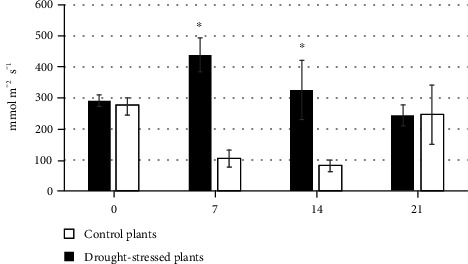
The response of stomatal conductance to moisture deficiency. Control and drought-stressed cv. Ikram plants were assayed after 0, 7, 14, and 21 d of treatment. Asterisks (∗) indicate significant differences of the drought-stressed plants from the control plants, according to Student's *t*-test (*p* ≤ 0.05).

**Figure 7:**
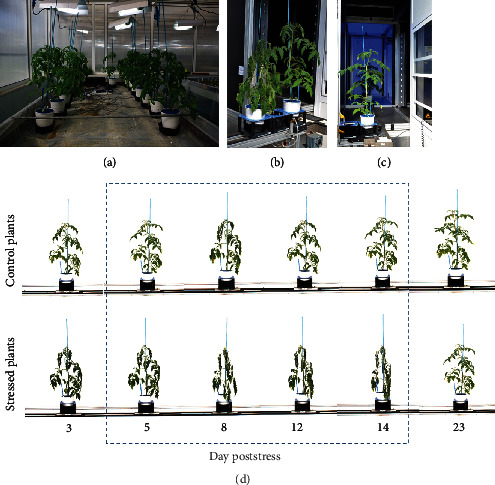
High-throughput phenotyping experiment: (a) plants implanted with a bioristor; (b, c) drought-stressed and well-watered plants during the process of image acquisition; (d) the plants monitored continuously with a bioristor and scanned every other day with the Scanalyzer over a period of 23 days.

The validation of the bioristor as a tool permitting the early detection of drought stress allowed a more detailed analysis conducted in the main experiment, in which four drought-stressed and four well-watered cv. Ikram plants were continuously monitored over a period of 23 d under controlled conditions (Figure 5). Once again, the slope of the NR parameter was used to define a number of phases. The PI phase followed the implantation of the sensor; during PII, there was an initial (days 2-4) sharp fall in NR as the intensity of the drought stress increased, but over the subsequent 4 d, NR recovered somewhat (DA) but with higher extent as for the Red Setter cultivar. The stress treatment had a strong effect on stomatal conductance, a widely used indicator of drought stress [31], over the period 7-14 d after the withholding of water reducing it by 4-5-fold in comparison to the well-watered plants (Figure 6). Reductions in stomatal conductance not only reduce transpirational water loss from the leaf but also constrain their photosynthetic activity, due to the limitation imposed on gas exchange [32]. An equivalent, although less pronounced, stomatal conductance response was, in retrospect, also recognized in the cv. Red Setter plants monitored in the pilot experiment, suggesting the existence of genotypic variation for the response. However, this defence mechanism failed as the stress period was prolonged, as was shown by the resumed fall in NR over days 7-9, after which the parameter remained at a low but stable level through to 23 d. A slight increase in the NR was observed corresponding to the limited irrigation operated (days 10-16). PIII was initiated upon the plants' rewatering, during which period NR increased to a level which was maintained through to the end of the experiment. This level was, however, much lower than the baseline obtained at the start of the treatment, reflecting a degree of irreversible damage caused by the stress (Figure 7(d)). Overall, the conclusion was that a stress response was detectable through the negative trend of the NR parameter already within the first 30 h following the withholding of water.

**Figure 8:**
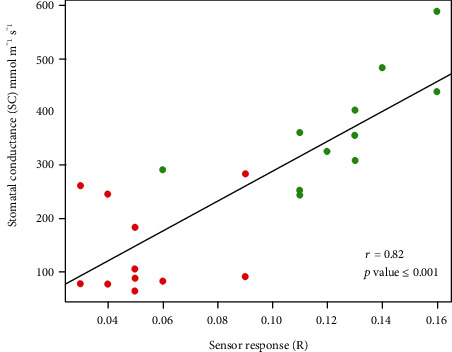
Scatter plots of the sensor response (*R*) and stomatal conductance (SC) measured on control plants (green) and drought-stressed plants (red). The scatter plot and linear regression displayed indicate a strong correlation between the two variables, with a correlation coefficient of *r* = 0.88. *p* < 0.001 indicates the statistical significance level of the observed correlation.

The ability of the bioristor to monitor physiological mechanisms strictly related to the changes of ion concentration in the plant sap in relation to the transpiration stream is strongly supported by the correlation analysis performed. In fact, a strong and highly significant correlation between the sensor response (*R*) and the stomatal conductance (SC) (*r* = 0.82, *p* value < 0.001; Figure 8) was observed, highlighting also a clear separation of the stressed and nonstressed samples indicating a strong influence of DS on the *R* value.

Moreover, to further demonstrate the ability of the bioristor in revealing changes in the concentration of the ions identified as the main players in the drought stress response, the transfer characteristics of the sensor response were measured, in vitro, using different concentrations of sodium (Na^+^), potassium (K^+^), calcium (Ca^2+^), and magnesium (Mg^2+^) salts. The analysis of the sensor response confirms the ability of the bioristor to detect changes in all the tested ions (Supplementary Fig. 2).

### 3.2. The Automated Monitoring of the Phenotypic Response to Stress

**Figure 9:**
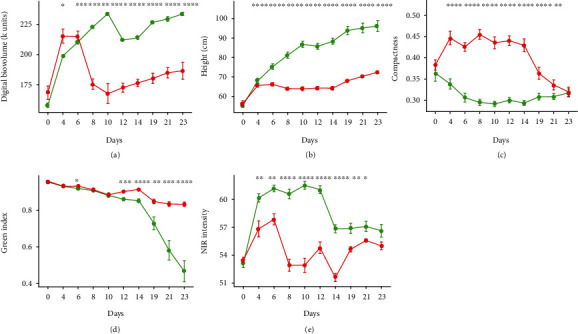
Indices derived from the Scanalyzer experiment imaging data on control plants (green) and drought-stressed plants (red): (a) digital biovolume, (b) plant height, (c) plant compactness, (d) green index, and (e) NIR intensity. Asterisks indicate significant differences of the drought-stressed plants from the control plants according to ANOVA (^∗∗∗∗^0 < *p* < 0.0001, ^∗∗∗^*p* ≤ 0.001, ^∗∗^*p* ≤ 0.05, and ^∗^*p* ≤ 0.01).

In the main experiment, imaging was used to monitor the phenotypic response to the stress treatment (Figures 7(a) and 7(b)). The chosen indices involved four based on RGB images (digital biovolume (Figure 9(a)), plant height (Figure 8(b)), plant compactness (Figure 9(c)), and green index (Figure 9(d))) and one based on NIR images (hydration index (Figure 9(e)). The growth of the plants, as indicated by their biovolume, height, and compactness, their greenness, and their hydration status were all strongly affected by the drought treatment. Changes in plant compactness and height were clearly visible, while the green and NIR-based indices first became detectable after 6-8 d (Figure 9). Biovolumes fell markedly over the first 8 d of the stress and were not recovered following rewatering (Figure 9(a)). The stress triggered severe wilting (Figures 7(b) and 7(d)); within 6 d of the withholding of water, the height of the stressed plants was 15% lower than that of the well-watered plants (Figure 9(b)). Plant compactness differed significantly between the treated and control plants within 4 d of the withholding of water; the stressed plants lost turgor, which steadily increased their compactness over the period 4-14 d; a gradual recovery occurred following rewatering and the compactness reached the level shown by the controls at the end of the experiment (Figures 7(d) and 9(c)). A similar trend was observed for the NIR intensity. For the control plants, the NIR-based index rose strongly over the first 6 d; this was also the case, although less markedly, for the stressed plants. For the latter, the index fell between days 7 and 10, until emergency irrigation was supplied to prevent plant death; the level was fully restored by rewatering carried out on day 16. The green index of the stressed plants fell slightly over the initial 12 d following the withholding of water, then remained steady, in contrast to the response of the control plants, which comprised a continuous fall over the whole measurement period (Figure 9(d)).

To confirm the suitability of the bioristor to complement the image-based high-throughput phenotyping techniques, we performed a Pearson correlation analysis between the digital biovolume as the index of drought stress [33] and the in vivo bioristor sensor response.

**Figure 10:**
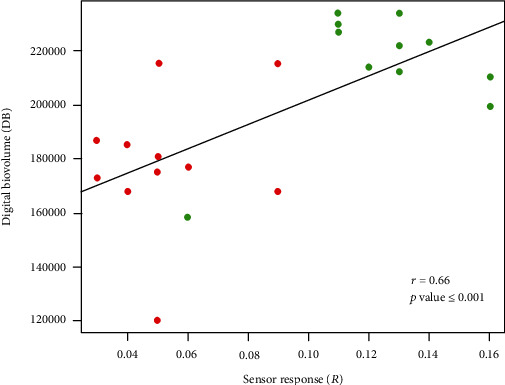
Scatter plot of the sensor response (*R*) and the digital biovolume (DB) measured on control plants (green) and drought-stressed plants (red). The scatter plot and linear regression displayed indicate a good correlation between the two variables, with a correlation coefficient of *r* = 0.66. *p* < 0.001 indicates the statistical significance level of the observed correlation.

A good and significant correlation (*r* = 0.66, *p* value < 0.001; Figure 10) was observed between the two variables, firstly confirming the occurrence of the drought stress and secondly supporting the suitability of the bioristor to monitor *in vivo* the drought stress profile in plants.

A comprehensive correlation analysis of all image-based index and manual-based measurements allowed us to observe a high correlation between the sensor response and those parameters linked with the transpiration process and the water use efficiency (SC and DB) and to exclude, at least in this experiment conditions, a direct correlation with the NIR intensity (NI), the green index (GI), and compactness (*C*, Supplementary Fig. 3).

In addition, to evaluate the overall phenotypic profile and distinguish plants of different agronomic groups, we performed a principal component analysis (PCA) using the “prcomp” function of R (https://cran.r-project.org/doc/FAQ/R-FAQ.html#Citing-R). Data were represented as a biplot (R package factoextra [34]), evaluating the compactness (C), green index (GI), stomatal conductance (SC), sensor response (*R*), NIR intensity (NI), and digital biovolume (DB) as variables.

**Figure 11:**
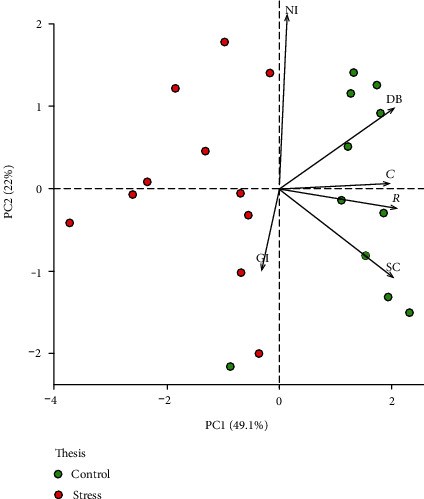
Biplot showing the PCA results. The first two PCs display 71.1% of the total phenotypic variation observed in 11 days of drought stress. The component scores (shown in points) are coloured according to the agronomic groups (red, drought-stressed plants; green, control plants). The component loading vectors (represented in lines) were superimposed proportionally to their contribution. *C*: compactness; GI: green index; SC: stomatal conductance; *R*: sensor response; NI: NIR intensity; DB: digital biovolume.

The first two components explain 71.1% of the variability (Figure 11). The first PC (PC1) explains almost a half (49.1%) of the phenotypic variation, which perfectly separated stressed plants from control plants. Stomatal conductance (SC) and digital biovolume (DB) have large positive loading on the PC1. The regularly irrigated controls and the drought-stressed plants are well separated in the biplot indicating the efficacy of the drought treatment.

## 4. Discussion

Plants commonly experience periods of moisture deficiency in the course of their life cycle [35]. Drought stress therefore represents a critical constraint over the productivity of crops [3]. An ability to detect drought stress before it causes irreversible damage is important for crop management and water savings and would also be valuable as a means of selecting varieties more resilient to the stress. Conventional phenotypic assays for drought tolerance are both labour intensive and imprecise, so the development of automated phenotyping platforms represents a promising advance [14]. Current platforms combine the robotic handling of plants with sensors and high-end computing to capture high-resolution, highly precise data in a high-throughput mode [14]. Their ability to collect data in real time in a nondestructive manner allows for potential insights to be gained into the temporal response of large numbers of plants to a particular treatment [9]. Moreover, an image-based index (RGB, NIR, and FLUO) is increasingly used to study the plant defence response mechanisms upon drought stress both in controlled conditions [3, 9, 29, 33, 36–38] and in the open field [39]. The development of both proximal and remote effective sensors has been accelerating of late, but there remains a lack of simple-to-operate and affordable sensors which can be implanted within the plant. The bioristor utilized here represents a model technology for generating *in vivo* high-resolution data of relevance to the plants' physiological status in real time and continuously. The bioristor was able to detect for the first time in vivo the onset of drought stress within 30 h of the withholding of water, and this is extremely relevant in terms of water use sustainability in the open field. In addition, its capacity to monitor the plants' physiological status on a continuous basis, rather than relying on sampling at a series of discrete time points, should greatly enhance the data's value in the context of understanding how plants respond to drought stress.

The breeding of crop varieties able to cope with the predicted changes in climate demands detailed knowledge of the mechanisms underlying the tolerance of plants to water deficiency and their ability to escape the stress. Changes in the composition of the xylem sap are thought to represent a major component of the drought defence machinery. Moisture deficiency inevitably reduces a plant's water content, thereby altering both the concentration of solutes in its transpiration stream and the mass of solutes exported from its roots at a given time [40].

Several reports have described the changes in nutrient and ion uptake and transport in plants during the occurrence of drought stress. In particular, a reduction in nutrient uptake by the roots partially due to the reduction in soil moisture was observed, which causes a decreased rate of nutrient diffusion from the soil matrix to the absorbing root surface [41] and translocation to the leaves [42, 43]. During stress, a reduction in the general mineral accumulation [43] in the plant tissues together with a general low nutrient availability in the soil and lower nutrient transport in plants was also reported [5, 41]. Stomatal closure is also a known mechanism of drought resilience that reduces transpiration, the nutrient transport from the roots to the shoot, and causes an imbalance in active transport and membrane permeability, resulting in a reduced absorption power in the roots [41, 44–46]. A direct measurement of the plant sap content during the drought stress also demonstrated that the amount of K^+^ ions gradually decreased in the xylem sap of maize plants [5].

The bioristor has been designed to detect the movement and concentration of electrolytes through the vascular tissues. Its application in the context of tomato plants exposed to drought stress has shown that changes in both the composition and the concentration of key solutes occurred within 30 h of the withholding of water. Xylem flux, transpiration rate, solubilisation, and translocation of solutes are all negatively affected by drought stress in a range of plant species [1, 5, 47–49], including tomato [1, 50]. The present data support the hypothesis that, as a result of the forced reduction in the transpiration rate, the early phase of the plant defence response includes a reduction in the concentration of ions in the xylem sap [5]. The integration of the bioristor data with image-based analyses has provided insights into the timing of the tomato phenotypic and physiological response, as well as suggesting that at the start of the drought stress response, a change in the transport, allocation, and production of metabolites and ions occurs within the plant, which acts as a signal for stomatal closure and the subsequent decrease in transpiration.

The high correlation coefficient between the sensor response (*R*), stomatal conductance (SC), and digital biovolume (DB) together with the PCA analysis confirms the hypothesis that the bioristor is able to detect ions and molecules related to the drought stress and, in particular, those dissolved and transported through the transpiration stream, thus efficiently detecting the occurrence of drought stress immediately after the priming of the defence responses.

A difference in the extent and timing of a possible drought avoidance (Figures 2 and 4) was observed between the two cultivars tested, opening new perspectives for the use of a bioristor as a tool for genotype selection in prebreeding.

## 5. Conclusions

The present experiments represent a breakthrough in the use of *in vivo* sensing technology in tomato. The data generated have shown how dynamic changes in the chemical composition of the sap in the xylem occur in drought-stressed tomato plants, with these changes becoming detectable within the first 30 h of water deprivation. The key characteristics of the bioristor, namely, its ability to continuously monitor aspects of the plant physiological status, its minimal invasiveness [17], its low cost, and its ease of generating readable data, suggest that the bioristor would represent a valuable tool in the context of precision agriculture and high-throughput drought response phenotyping. Moreover, its potential ability to differentiate the response of two cultivars differing from one another with respect to drought tolerance could be exploited as a selection tool for breeding more drought-resilient tomato cultivars. Among our current research priorities is the extension of the bioristor's ability to detect specific compounds of particular relevance to the plant's drought response. Much of the climate change impact on agriculture is mediated through water, and considering that agriculture is currently withdrawing 70% of the fresh water available in the planet, the application of the bioristor in open fields may represent a real innovation for increasing the water balance efficiency in agriculture addressing one of the UN sustainable development goals.

## Data Availability

The bioristor raw data and the images acquired with the Scanalizer platform are available upon request to the authors.

## Supplementary Materials

Supplementary 1 Supplementary Fig. 1: The bioristor biosensor. (A) Bioristor is an organic electrochemical transistor (OECT) based on two textile fibers functionalized with PEDOT:PSS acting as a channel (the upper) and as a gate (the lower); (B and C) example of the bioristor inserted in the tomato plants. (D) Bioristor holder.

Supplementary 2 Supplementary Fig. 2: Transfer characteristics of the sensor response measured using different concentrations of sodium (Na^+^), potassium (K^+^), calcium (Ca^2+^), and magnesium (Mg^2+^) salts expressed as the sensor response (*R*), where *I* is the off current (measured for gate voltages, *V*_g_ ≠ 0V) and *I*_0_ is the on current (measured for *V*_g_ = 0V).

Supplementary 3 Supplementary Fig. 3: Correlation of the acquired physiological and morphological measurements including the bioristor response (*R*), image-derived data (DB: digital biovolume; GI: green index; NI: NIR intensity; *C*: compactness), and manually determined stomatal conductance (SC). Red dots indicate stressed plants; green dots indicate control plants.
