# Supplementary figures and images for: In Vivo Phenotyping for the Early Detection of Drought Stress in Tomato
Source: Plant Phenomics. 2019 Nov 27;2019:6168209. doi: 10.34133/2019/6168209 (PMC7706337; doi:10.34133/2019/6168209)

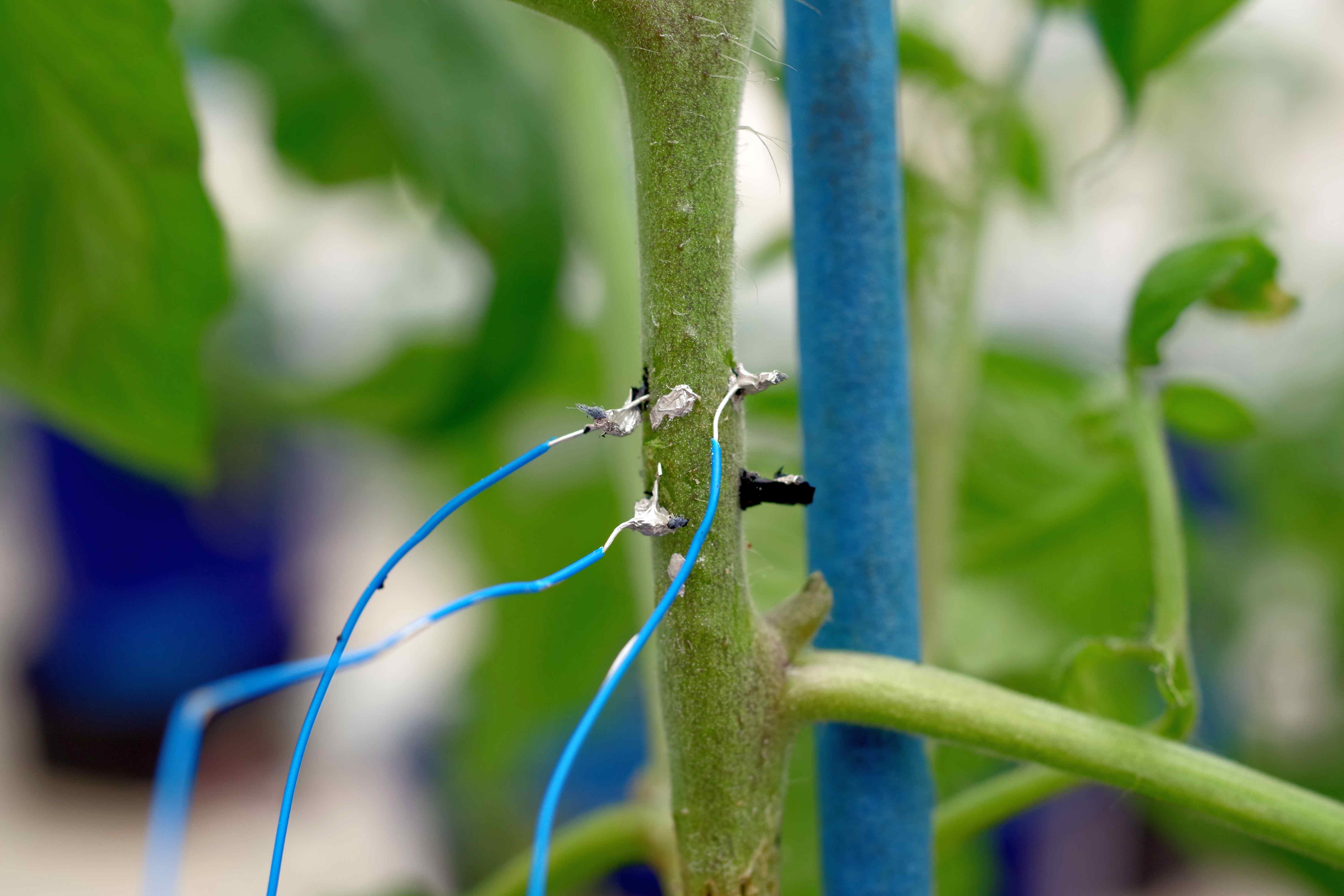

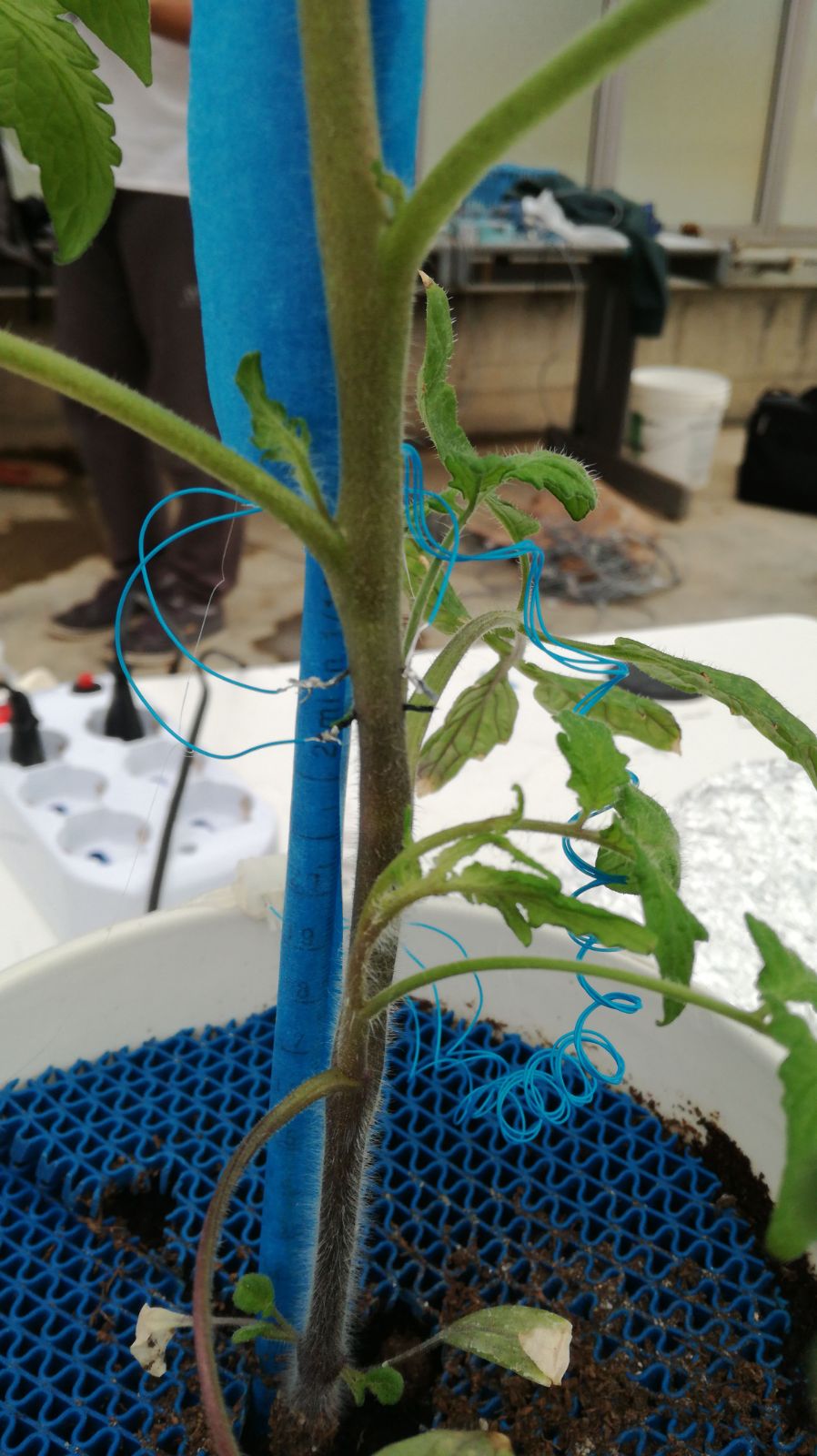

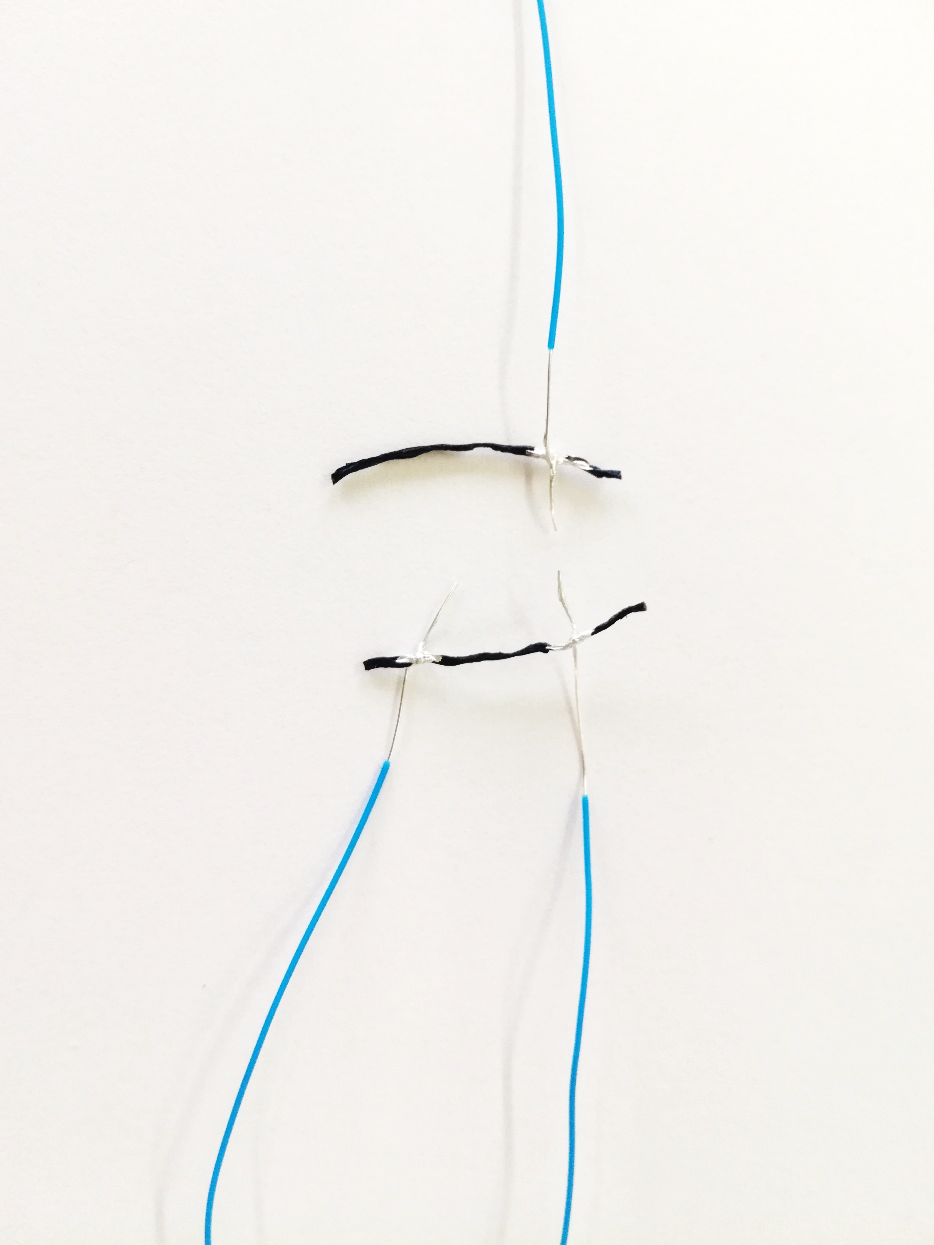

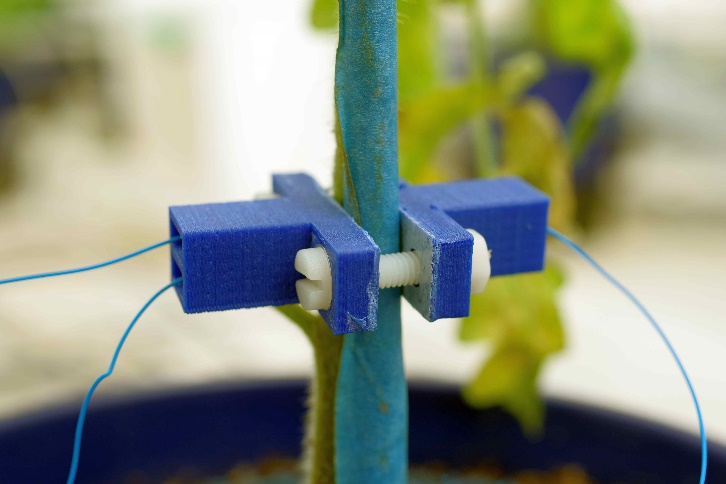


A)

B)

C)

channel

gate

electrical

wire

D)

Supplement: Supplementary 1 — Supplementary Fig. 1: The bioristor biosensor. (A) Bioristor is an organic electrochemical transistor (OECT) based on two textile fibers functionalized with PEDOT:PSS acting as a channel (the upper) and as a gate (the lower); (B and C) example of the bioristor inserted in the tomato plants. (D) Bioristor holder. [file 6168209.f1.docx]
